# Supplementary material for: Development of chemokine network inhibitors using combinatorial saturation mutagenesis
Source: Commun Biol. 2025 Apr 3;8:549. doi: 10.1038/s42003-025-07778-6 (PMC11969024; doi:10.1038/s42003-025-07778-6)
Supplement: Supplementary file 4 — Supplementary Data 2 [file 42003_2025_7778_MOESM4_ESM.zip › SupplementaryData3/scripts/xattune_ic50.tar!/./script/analysis/JK/JK_R31_E61_RA_HD2CM304_THP1_plot.pdf]

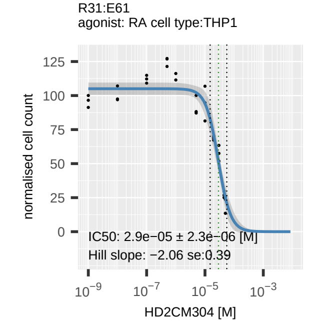
R31:E61
agonist: RA cell type:THP1
125
100
75
50
25
0
normalised cell count
IC50: 2.9e−05 ± 2.3e−06 [M]
Hill slope: −2.06 se:0.39
10^{-9}
10^{-7}
10^{-5}
10^{-3}
HD2CM304 [M]
